# Higher risk of progressing breast cancer in Kurdish population associated to CDH1 -160 C/A polymorphism

**DOI:** 10.17179/excli2017-843

**Published:** 2017-11-06

**Authors:** Farzaneh Zarei, Mohammad Nazir Menbari, Bayazid Ghaderi, Mohammad Abdi, Zakaria Vahabzadeh

**Affiliations:** 1 Department of Biology, Sanandaj Branch, Islamic Azad University, Sanandaj, Iran; 2 Cellular and Molecular Research Center, Faculty of Medicine, Kurdistan University of Medical Sciences, Sanandaj, Iran; 3 Liver and Digestive Research Center, Kurdistan University of Medical Sciences, Sanandaj, Iran; 4 Department of Internal Medicine, Faculty of Medicine, Kurdistan University of Medical Sciences, Sanandaj, Iran; 5 Department of Clinical Biochemistry, Faculty of Medicine, Kurdistan University of Medical Sciences, Sanandaj, Iran

**Keywords:** breast cancer, CDH1, metastasis, polymorphism, prognosis

## Abstract

There is an increasing interest about studying possible effects of genetic polymorphisms and risk of cancer progression. E-cadherin (CDH1) involves in many important cellular processes including cell-cell interactions, cell development and genetic changes of this molecule has been associated with greater tumor metastasis. The present study was aimed to evaluate the possible role of CDH1 -160 C/A polymorphism as a potential risk factor for breast cancer in Kurdish population. This case-control study consisted of 100 breast cancer patients and 200 healthy controls. Clinicopathological findings of all individuals were reported and immunohistochemistry staining was carried out on tissue samples. The CDH1 -160 C/A genotype was determined by polymerase chain reaction- restriction fragment length polymorphism method (PCR-RFLP). CDH1 -160 C/A polymorphism was differently distributed between patient and control groups. The A allele of CDH1 -160 C/A polymorphism significantly increased in patients compared to controls. In addition we found that the A allele of this polymorphism might be a potential risk factor for progression of breast cancer in our studied population. Patients with A allele of CDH1 -160 C/A was in higher risk to progress invasive ductal carcinoma. The A allele was also correlated with high grade and stage IV and also with metastatic tumors in studied subjects. The CDH1 -160 C/A polymorphism is correlated with clinicopathologial findings of breast cancer patients. The A allele of CDH1 -160 C/A may be a risk factor for progression of breast cancer in Kurdish patients.

## Introduction

According to data released by WHO, breast cancer is one of the most important leading cause of death from cancer in different populations (Benson and Jatoi, 2012[5]). In Iran, studies show that 6160 women are diagnosed with breast carcinoma yearly (Mousavi et al., 2007[24], 2009[23]; Alizadeh Otaghvar et al., 2015[3]). In addition to environment factors, genetic status of patients has also very effective role in development of cancer. Besides, previous data have clearly proved that genetic variations can effect on developing certain type of a carcinoma, treatment and prognosis of cancer (Pharoah et al., 2004[25]). 

Given the importance of determining the association between genetic variations and progression of disease, our research group has begun studies in the past three years in this field. Our previous studies showed that single nucleotide polymorphisms (SNPs) in ATP-binding cassette, sub-family B (MDR1 or ABCB1), X-ray repair cross-complementing group 1 (XRCC1) and ATP-binding cassette sub-family G member 2 (ABCG2) might be probable risk factors for chronic lymphocytic leukemia (CLL), breast cancer and chronic myeloid leukemia (CML) (Maroofi et al., 2015[22]; Ghafouri et al., 2016[10]; Jalali et al., 2016[15]; Salimizand et al., 2016[26]). 

Cadherin 1 (CDH1) (16q22.1) is a tumor suppressor gene that encodes a calcium-dependent cell-cell adhesion glycoprotein named epithelial cadherin (E-cadherin or uvomorulin) (Kangelaris and Gruber, 2007[17]). E-cadherin involves in various cellular mechanisms including cellular morphology and differentiation, signaling system and polarity of the cells (Tepass et al*.*, 2000[28]; Gumbiner, 2005[14]). Data released by previous researches clearly raveled the anti-cancer effects of this gene and showed that genetic mutations of CDH1 are correlated to many types of cancer like gastric cancer, breast cancer, ovarian cancer, colorectal cancer and thyroid cancer (Ahmadi et al., 2013[2]; Govatati et al., 2014[12]; Li et al., 2014[19]). Among all polymorphisms of CDH1 gene, it seems that -160C/A (rs16260) SNP has a significant role on transcriptional activities (Li et al., 2000[20]; Jeanes et al., 2008[16]; van Roy and Berx, 2008[30]). There is paucity of studies with regard to -160C/A CDH1 SNP in breast cancer and there is no other study about association of this SNP with risk of BC in Iranian population. Furthermore, the previous studies had very conflicted results with regard to association between CDH1 SNPs and risk of cancers. In the case of breast cancer, the results are very inconsistent, although it seems that A allele of -160C/A SNP may be a potential risk factor for breast cancer. According to the above mentioned, further studies in this issue are much needed. The present study was directed to assess the role of -160 C/A CDH1 SNP as a possible risk factor in breast cancer and the association of this polymorphism with clinical and laboratory findings of BC patients in an Iranian population.

## Material and Methods

### Patients

The studied population included Kurd patients who are diagnosed as BC based on histopathological examination of breast tissue. The blood and tissue specimens were sampled between January 2012 to May 2015 from patients with suspected breast cancer who were admitted to Tohid Hospital, Kurdistan, Sanandaj, Iran and after pathologic examination positive results were considered as case and negative results as control group. Accordingly, a total of 100 people, age 47.13±8.4 years, were enrolled in patients group and 200 healthy age matched subjects were considered as controls (*p value* > 0.05). Written informed consent was obtained from all patients, and the study has been approved by the ethics committee of Kurdistan University of Medical Sciences. Subjects with a past history of other organ cancers were rolled out from the study. The criteria and system used for grading and staging the tumors were Scarf-Bloom-Richardson and TNM staging system for breast cancer, respectively (Elston, 2005[8]; Edge et al., 2010[7]). The follow-up time was a 24 months median (0-48 months) and the majority of chemotherapeutic agents consist of anthracyclines (doxorubicin and cyclophosphamide) and Paclitaxol. 

### Tissue preparation and immuno-histochemistry analysis assay

Tissue samples with positive results are considered for immunohistochemistry (IHC) investigation. Tissue preparation and IHC assay including estrogen receptor (ER), progesterone receptor (PR), and Ki67 and Her2/neu staining, are assessed according to our previous studies (Ghafouri et al., 2016[10]; Jalali et al., 2016[15]).

### Discrimination of -160 C/A CDH1 genotypes by polymerase chain reaction-restriction fragment length polymorphism (PCR-RFLP) method

Genomic DNA was extracted from whole blood using DNP^TM^ reagent (CinnaGen Inc, Tehran, Iran) according to our previous studies (Abdi et al., 2014[1]; Maroofi et al., 2015[22]; Amini et al., 2016[4]; Ghafouri et al., 2016[10]; Jalali et al., 2016[15]; Salimizand et al., 2016[26]). The -160 C/A CDH1 SNP were determined using PCR-RFLP. The PCR reaction was carried out in a final volume of 25 μL using PCR Master Mix kit (CinnaGen Inc, Tehran, Iran), 10 pmol of each primer with final concentration of 400 nM, and 100 ng DNA. Two primers were used to amplify a fragment of 328bp of CDH1 gene. CDH1 forward primer was 5′- TGATCCCAGGTCTTAGTGAG-3′, and CDH1 reverse primer was, 5′-AGTCTGAACTGACTTCCGCA-3′. The PCR conditions was: 5 min at 95 °C (initial denaturation), followed by 45 cycles of 95 °C for 30 s (denaturation), and 58 °C for 30 s (annealing) and 72 °C for 30 s using an Eppendorf Mastercycler (Eppendorf AG, Hamburg, Germany). In each PCR run, samples with no DNA template were used as negative controls. Amplified DNA fragments (328 bp) were cut by restriction enzyme BsteII (Jena Bioscience, Germany) for 30 min at 37 °C. The genotypes were determined by electrophoresis of DNA fragments generated after digestion (two bands: 218 and 110 bp for CC genotype, one band: 328 bp for AA genotype and three bands: 328 bp, 218 bp and 110 bp for heterozygous CA genotype).

### Statistical analysis

Data were analyzed by SPSS 16 (SPSS Inc., Chicago, IL, USA), and a Chi-square test was used to evaluate whether the alleles or genotype frequencies differ between studied groups. For 2×2 contingency tables, the odds ratio and its 95 % confidence interval were calculated for different genotypes and allele and also for clinicopathological findings and *p value* <0.05 was considered statistically significant.

## Results

A total of 100 breast cancer patients and 200 healthy controls participated in the study. There was not a statistically significant difference between case and control groups for age (47.13±8.4 and 46.8±7.3 years respectively, *p value*> 0.05). Most of cases (82 patients) recognized with invasive ductal carcinoma (IDC) and 12 patients with invasive lobular carcinoma (ILC). Tumor grading results determined 12 patients with low grade, 60 patients with intermediate and 28 cases with high grade. There were also higher frequencies for stage III and II (38 and 30 cases, respectively) followed by stage IV and I (17 and 15 patients, respectively). Most patients (88 cases) were undergoing chemotherapy regimen; surgery and radiotherapy were used for 60 and 59 patients, respectively. Besides, IHC results showed that 83 cases were ER positive, 76 PR positive, 48 Her2/neu positive and 54 patients were Ki67 positive.

Genotype distribution was in accordance with the Hardy-Weinberg Equilibrium for patients and controls (*p value*>0.05). There was a statically significant difference between studied groups for the CDH1 -160 C/A genotypes (*p value*=0.026) (Table 1(Tab. 1)); our results demonstrated that the rate of AA genotypes was higher in patients compared to healthy subjects and CC genotypes had also increased rate in controls (Table 1(Tab. 1)). Accordingly, the frequency of CC, CA and AA genotypes in patients and controls were 47 (47 %), 44 (44 %), 9 (9 %) and 131 (65.5 %), 65 (32.5 %), 4 (2 %), respectively. The patients with CA heterozygous genotype were significantly increased the risk of developing BC compared to CC genotype (OR= 1.8867, 95 % CI= 1.135-3.1339, Z statistic= 2.452, *p value*= 0.0142). In addition, the patients with mutant homozygous AA genotype had higher risk of progressing breast cancer compared to CC genotype with an OR of 6.2713 (95 % CI= 1.8440-21.3283, Z statistic= 2.940, *p value*= 0.0033). Besides, the CA and AA genotypes together also increased the risk of breast cancer (OR= 2.1409, 95 % CI= 1.3128-3.4915, Z statistic= 3.051, *p value*= 0.0023) compared to CC genotype. The A allele of CDH1 -160 C/A gene was higher in patients than controls (*p value*=0.0369) and it was also associated with breast cancer risk (OR= 2.0467, 95 % CI= 1.0544-3.9727, Z statistic= 2.117, *p value*= 0.0343) (Table 1(Tab. 1)).

Clinical, pathological and laboratory characteristics of patients were measured in different genotypes and the probability of being as a risk factor was evaluated for them. Table 2(Tab. 2) shows this evaluation. According to this table, patients who are diagnosed as Invasive ductal carcinoma (IDC) were found to have a significant rate of A allele compared with Invasive lobular carcinoma (ILC) patients (OR= 1.7675, 95 % CI= 1.0066-3.1037, Z statistic= 1.983, *p value*= 0.04) (Table 2(Tab. 2)). In addition, analysis of our data showed that there was a statistically significant number of A allele in patients with stage IV breast cancer compared with stage I patients (OR= 1.9412, 95 % CI= 1.0978-3.4324, Z statistic= 2.281, *p value*= 0.0226). The rate of A allele was also higher in high grade patients compared to low grade patients (OR= 2.1905, 95 % CI= 1.2172-3.9419, Z statistic= 2.616, *p value*= 0.0085). Finally, our results demonstrated that metastatic cases were found to have a significant frequency of A allele compared with non-metastatic patients (OR= 1.833, 95 % CI= 1.0456-3.2144, Z statistic= 2.116, *p value*= 0.0344). However, there was not a significant correlation between CDH1 -160 C/A SNP with protein expression in breast tumor tissues (Table 3(Tab. 3)). In addition, we did not find any statistically significant association between studied SNPs and age of individuals. No other parameter was found to be significantly associated with CDH1 -160 C/A polymorphisms (Table 2(Tab. 2)).

## Discussion

In the present study we showed significant association between CDH1 -160C/A polymorphism and breast cancer risk in Kurdish women. E-cadherin plays an important role in cell activities such as differentiation, signaling and adhesion (Frixen et al., 1991[9]). Down-regulation of CDH1 promotes malignant transformation, tumor invasion and metastasis. Diminishing of CDH1 expression is proposed as an important factor in the pathogenesis of breast cancer (Li et al., 2014[19]). Previous reports showed that E-cadherin is definitely suppressing the invasion of cancer cells to distant sites (Li et al., 2014[19]). It has proved that the cell-cell adhesion molecule E-cadherin involves in maintenance of cell integrity has a supporting role for epithelial development and organization (Frixen* et al.*, 1991[9]).

On the other hand, Govatati et al. (2012[13]) revealed that the E-cadherin expression considerably depends on the type of the CDH1 gene polymorphisms. They showed that the -160A, -347GA and +54T alleles of CDH1 genes can reduce the expression of CDH1 gene. Based on *in vitro* studies, the expression of CDH1 gene decreased by 68 % in cells with mutant homozygous AA genotype of CDH1 -160 C/A SNP compared with the C allele (Li et al., 2000[20]). Therefore, A allele can be considered as a strong genetic factor in patients with high invasive or metastatic tumors. The -160 locus is located at the upstream of the transcriptional start site of CDH1 gene. This region is near to several cis-acting elements, for example E boxes, CAAT box, SP1-binding site (Giroldi et al., 1997[11]). Therefore, genetic variation in this site potentially effects on the expression of CDH1 gene. 

There is a controversy with regard to the role of CDH1 -160 C/A SNP and risk of malignancy. Although previous studies showed the association of CDH1 -160C/A SNP with different types of cancers (Wang et al., 2008[31]; Tipirisetti et al., 2013[29]), some studies disagreed with this hypothesis (Lei et al., 2002[18]; Cattaneo et al., 2006[6]). Especially in breast cancer, the number of the studies confirmed the role of CDH1 -160C/A SNP in progression of cancer is equal to those that reject this role. 

Cattaneo et al. (2006[6]) showed that there was no association between CDH1 -160C/A and risk of breast cancer (OR=1.35, 95 % CI= 0.84-2.17) among the Italian population. In another study, Tipirisetti et al. (2013[29]) investigated the association between CDH1 SNPs and breast cancer risk in south Indian women. The results of this study showed that there were significantly higher frequencies of -160A/A genotypes (*p value*= 0.038) and 160A alleles (*p value*= 0.046) in patients compared to controls. They suggested that the CDH1 -160C/A polymorphism may be proposed as a genetic risk factor for breast cancer in south Indian women. In a recent study, Shabnaz et al. (2016[27]) revealed that CDH1 -160 C/A SNP is a potential risk factor for breast cancer among Bangladeshi women. Recently, a meta-analysis depicted that CDH1 -160 C/A SNP might contribute to breast cancer susceptibility. However, the authors stated that further studies with large number and different ethnicity are needed to verify their results (Ma* et al.*, 2016[21]). In line with Shabnaz and Tipirisetti studies, our investigation showed that the A allele of CDH1 -160 C/A polymorphisms might be a potential risk factor for progression of breast cancer, although, our results were not in agreement with Cattaneo report. This controversy may be a result from different selection of studied subjects or variability in genetic and ethnic factors of studied populations. Our investigation had some new findings; although we did not find a significant correlation between AA/CA genotypes of CDH1 -160 C/A SNP with histopathological indices including ER, PR, Her2/neu and Ki67, we proved that the A allele is a risk factor for progression to stage IV and high grade. We also showed that the association of the A allele of CDH1 -160C/A SNP with breast cancer metastasis is statistically significant. 

In conclusion, our results show CDH1 -160 C/A is associated with increased breast cancer risk in Kurdish women. In addition, the presence of A allele of CDH1 -160 C/A polymorphism may play an important role in metastasis of breast cancer. Additional, larger population-based studies as well as functional evaluation of the variants are necessary to confirm our findings.

## Acknowledgements

The authors wish to thank all patients and health stuffs who participated in this study. Financial support from Kurdistan University of medical sciences is highly appreciated.

## Disclosure of potential conflicts of interest

Mrs F Zarei declares no potential conflicts of interest with respect to the research, authorship, and/or publication of this article. Mr M-N Menbari declares that he has no conflict of interest. Dr B Ghaderi declares no potential conflicts of interest with respect to the research, authorship, and/or publication of this article. Dr M Abdi has received research grants from the Ministry of Health and Medical Education (MOHME) of Iran. Dr Z Vahabzadeh declares that he has no conflict of interest. 

## Ethical approval

All procedures performed in studies involving human participants were in accordance with the ethical standards of the ethics committee of Kurdistan University of Medical Sciences and with the 1964 Helsinki declaration and its later amendments or comparable ethical standards.

## Informed consent

Informed consent was obtained from all individual participants included in the study.

## Funding source

This work was supported by the Ministry of Health and Medical Education (MOHME) of Iran (Grant/Award Number: '1395/306').

## Financial disclosure

The author has no financial relationships relevant to this article to disclose.

## Author contributions

All authors contributed equally in this work.

## Figures and Tables

**Table 1:**
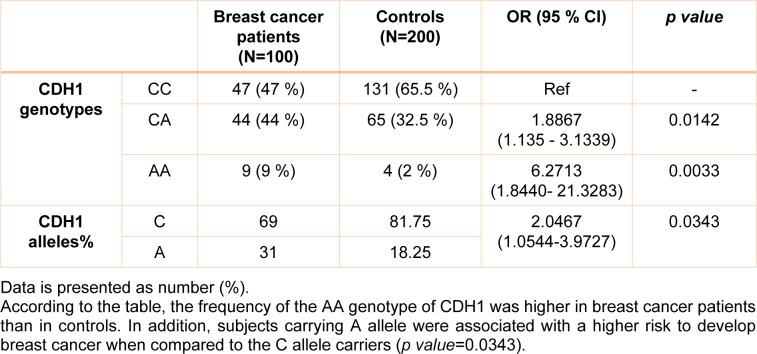
Genotypes and alleles frequencies of CDH1 -160 C/A SNP in patient and control groups

**Table 2:**
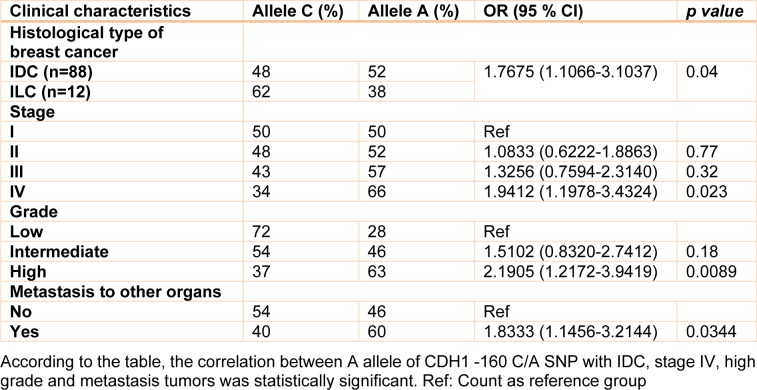
Correlation of A allele of the CDH1 -160 C/A SNP with clinical characteristics of the patients

**Table 3:**
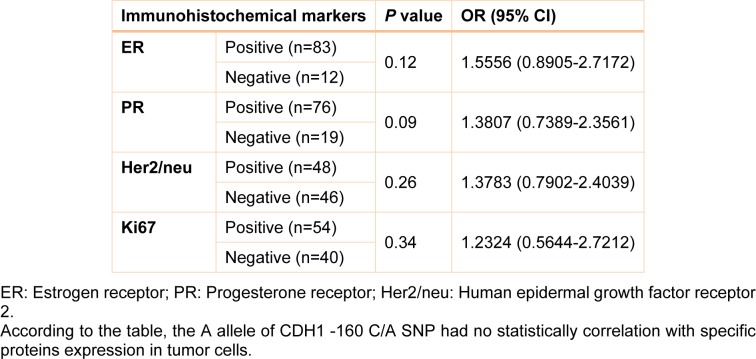
Association between A allele of CDH1 -160 C/A SNP with protein expression in breast tumors
